# The associated factors to endometrial cavity fluid and the relevant impact on the IVF-ET outcome

**DOI:** 10.1186/1477-7827-8-46

**Published:** 2010-05-14

**Authors:** Rong-Huan He, Hui-Juan Gao, Ya-Qiong Li, Xiao-Ming Zhu

**Affiliations:** 1 Department of Reproductive Endocrinology, Women's Hospital, Zhejiang University School of Medicine, Hangzhou, Zhejiang, China

## Abstract

**Background:**

Endometrial cavity fluid (ECF) is a fluid accumulation within the endometrial cavity. The significance of ECF remains unclear during the program of in vitro fertilization-embryo transfer (IVF-ET). The aim of the present study was to investigate the associated factors to ECF, visualized through ultrasound at the day of oocyte retrieval, and the relevant impact on the outcome of IVF-ET.

**Methods:**

From the clinical data of 1557 infertility patients for IVF-ET program, 46 ECF patients were retrospectively selected as the ECF group; and another 134 patients with a bilateral salpingectomy and without ECF, selected as the control group. The demographics and the outcome of IVF-ET were compared between the two groups.

**Results:**

The incidence of ECF was 2.95% (46/1557). Over half (28/46, 60.87%) of ECF patients had tubal infertility. Only 12 Of 46 ECF patients (26.09%) had visible hydrosalpinx on ultrasonography before ovarian stimulation. The cycle cancellation rate (4/46, 8.69%) of ECF group was not significantly higher than that of the control group (6/134, 4.48%; P > 0.05). Reasons for cycle cancellation in both groups were all the high risk of ovarian hyperstimulation syndrome (OHSS). No significant difference was found in clinical pregnancy rate between the patients with their ECF <3.5 mm in the anterior-posterior diameter (APD) and the control group (35.48% versus 30.47%; P > 0.05). No clinical pregnancy was found among those patients with their ECF equal or higher 3.5 mm in APD.

**Conclusions:**

It was tubal infertility, not hydrosalpinx, which was related to the development of ECF. Excessive ECF (equal or higher 3.5 mm in APD) at the day of oocyte retrieval would have a negative impact on the outcome of IVF-ET.

## Background

Endometrial cavity fluid (ECF) is a fluid accumulation within the endometrial cavity. Although the importance of endometrial thickness during in vitro fertilization (IVF) cycles has been documented much, the significance of ECF remains unclear [1-7]. ECF after ovarian stimulation and before embryo transfer (ET) is not a common complication in IVF, but excessive uterine fluid is detrimental to embryo implantation [1,2]. It was hypothesized that embryonal apposition may not occur when a fluid layer is overlaying the endometrium. A few studies involving low numbers of patients have been reported on the development of ECF in patients undergoing assisted reproductive techniques [1-7], some of them claimed that the development of ECF was related to hydrosalpinx [3,6,8,9] and some others, not[5,7]. The cycles with ECF were considered by most researchers to have low implantation and pregnancy rates as well as a high incidence of cycle cancellation [1,2,5].

In our clinical practices, we found many cases with ECF had their babies successfully through their IVF program. Is there a defining value of ECF, above which, the negative impact of ECF on the outcome of IVF-ET is great; and under which, the negative impact might be little?

The aim of the present study was to investigate the associated factors to ECF, visualized through ultrasound at the day of oocyte retrieval, and the relevant impact on the outcome of IVF-ET. In the present study, a retrospective investigation in this aspect was shown based on the clinical data of infertility patients enrolled for the IVF program. ECF patients were selected and sub-grouped based on the maximal fluid anterior-posterior diameter (APD) between the anterior and posterior endometrial linings in a sagittal view of uterine cavity at the day of oocyte retrieval; and non-ECF ones selected as the control group. The demographics and the outcome of IVF-ET were compared between groups.

## Methods

### Patients

The study was approved by the Review Board of Women's Hospital, Zhejiang University School of Medicine. The clinical data of 1557 cycles of infertility patients that enrolled in our IVF program between March 1, 2006 and February 28, 2007 was retrospectively reviewed. 46 patients were selected as the ECF group, in which there was a fluid accumulation equal or higher1.0 mm in the anterior-posterior diameter (APD) in the uterine cavity of each patient at the day of oocyte retrieval; and another 134 ones with a bilateral salpingectomy selected as the control group, in which no fluid accumulation was detected in their uterine cavities at the day of oocyte retrieval.

The cervical stenosis of each selected patient has been eliminated by the uterine exploration routinely carried out at the initiation of each IVF program. No other causes recorded influencing fertility, such as endometriosis, leiomyoma, etc., were found in each of the selected cases. Cycles that involved the use of frozen embryos, donor oocytes, or assisted hatching were excluded. All pregnancies were confirmed by the rising serum β-HCG levels and the gestational sacs identified by transvaginal sonographic examination.

### IVF-ET procedures

All patients underwent pituitary desensitization followed by controlled ovarian hyperstimulation. Briefly, GnRH-agonist suppression in a standard long protocol by s.c. injections of triptorelin acetate (Decapaptyl, Ferring, Germany), 0.1 mg/day started on Day 21 of the prior menstrual cycle. Ovarian stimulation was then initiated with rFSH (Gonal-F; Serono, Aubonne, Switzerland) and/or HMG (Pergonal^®^; Serono Menotropin Livzon). Follicular growth was monitored by assay of estradiol (E2) levels and ultrasonography. When at least two leading follicles reached to a diameter >16 mm, HCG (Pregnyl; Serono, Aubonne, Switzerland) was administered at a dose of 5000-10000 IU. Oocytes were retrieved by transvaginal ultrasound-guided follicular aspiration 34-36 h after HCG injection. The infertility due to severe male factors was treated with the intracytoplasmic sperm injection, while standard IVF techniques were used for others. All patients had at least one good-quality embryo for transfer on the second or third day after oocyte retrieval. The embryos were evaluated by a scoring system named cumulative embryos score which was based on blastomere number and fragmentation pattern [10]. The number of embryos transferred varied between patients. All ETs were performed with an Edward-Wallace catheter (k-OPSD-1635-ET, COOK, Queensland, Australia) under ultrasound guidance. Luteal support consisted of 50 mg progesterone in oil administered i.m. daily in patients who had ET performed.

### Ultrasonography examination of ECF

Sonographic examinations were performed using an Ultramark^® ^9 HDI (Advanced Technology Laboratories, Bothell, WA, USA) with a 5 MHz multi-frequency transvaginal probe. The endometrium was scanned sagittally along the mid-line axis of the uterus. Alterations in the endometrial thickness and echogenic pattern/structure were recorded respectively during gonadotrophin administration, on the day of oocyte retrieval and on the day of ET.

**Figure 1:**
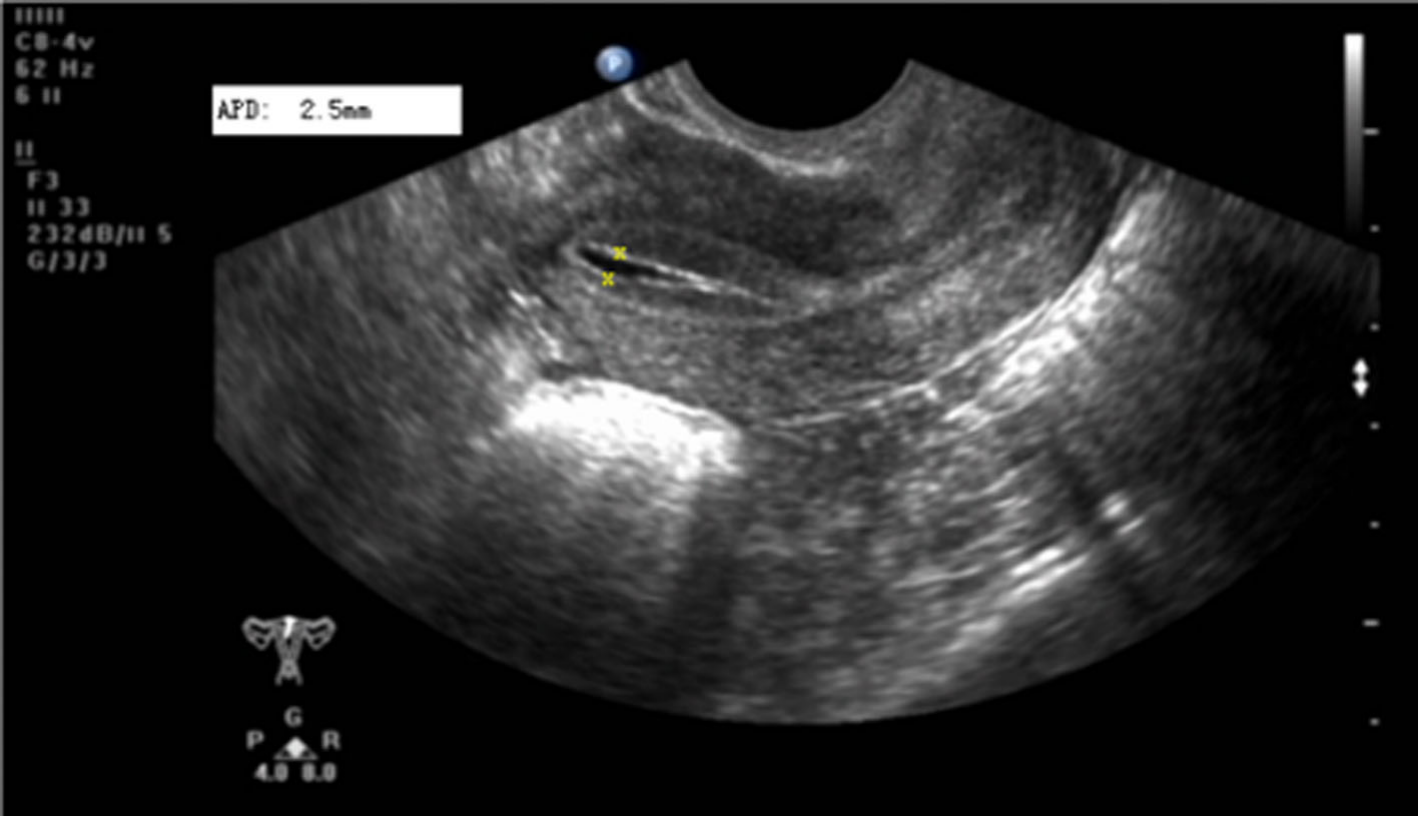
**Endometrial cavity fluid (ECF)**. Fluid accumulation in the uterine cavity detected by transvaginal ultrasound in a sagittal view (A--P diameter 2.5 mm) in a patient on the day of oocyte retrieval.

Fluid accumulation within the uterine cavity was defined as an echolucent ring configuration distended by a certain amount of fluid and detected by transvaginal ultrasound. Here, APD, the maximal fluid diameter between the anterior and posterior (A-P) endometrial linings in a sagittal view of uterine cavity, was used to assess the degree of fluid accumulation (Fig. 1). In cases of fluid accumulation, the thickness of endometrium was measured by subtracting the maximal fluid diameter from the maximal distance between the opposing myometrial/endometrial interfaces. The maximal fluid diameter and the surrounding endometrial thickness were used as indices for analysis.

The charts of all patients undergoing IVF treatment were searched for the presence of fluid accumulation inside the uterine cavity on the day of oocyte retrieval. That fluid might have disappeared or persisted until the end of the cycle, but never aspirated. The cases whose fluids were detected only during the period of ovarian stimulation were excluded.

### Statistics

Continuous data were presented as the mean ± SEM. Comparisons between groups were performed using the Mann-Whitney U-test or Student's t-test as appropriate. The statistical test to analyze the reproductive outcomes was done using chi-square analysis. A P-value less than 0.05 was considered statistically significant.

## Results

From a total of 1557 cycles involving 1546 patients, ECF was detected in 46 cycles of 46 different women, with an incidence of 2.95% (46/1557). In all of these cases, the endometrium had been evaluated as normal before and during ovarian stimulation. All the fluid accumulation was emerged at the time of oocyte retrieval, after HCG administration. The range of ECF in APD was from 1.0 mm ~10.0 mm.

Over half (28 out of 46 cycles, 60.87%) of the women who showed ECF during IVF-ET cycles had tubal infertility. Only 12 of the 46 ECF cases (26.09%) had visible hydrosalpinx on ultrasonography before ovarian stimulation. Among the other 18 cases of ECF women with non-tubal infertility, nine had endometriosis as the infertility cause for IVF, six the male infertility factors, two the unexplained infertility and one the polycystic ovarian syndrome (PCOS).

The demographic, clinical parameters and cycle outcomes of patients of the ECF group and control group are shown as Table 1. Both groups were comparable in terms of background characteristics. No difference was detected in implantation rate (12.62% versus 17.97%; X^2 ^= 1.529, P > 0.05), clinical pregnancy rates (26.19% versus 30.47%; X^2 ^= 0.279, P > 0.05) and the cycle cancellation rate (8.69% versus 4.48%; X^2 ^= 1.161, P > 0.05) between the two groups. Reasons for cancellation of the corresponding cycles in both groups were all the high risk of ovarian hyperstimulation syndrome (OHSS).

**Table 1:** Patients' demographics and cycle parameters

	ECF Group (n = 46)	Control Group (n = 134)	p --value (95% CI)
Age (years old)	31.5 ± 0.5	31.8 ± 0.1	>0.05
Duration of infertility (years)	5.4 ± 0.4	5.5 ± 0.1	>0.05
Baseline FSH (mIU/ml)	7.8 ± 1.2	7.6 ± 1.6	>0.05
Baseline LH (mIU/ml)	5.0 ± 1.5	5.1 ± 1.7	>0.05
E_2 _level on the day of HCG (pg/ml)	2188.6 ± 1546.9	2210.5 ± 1125.8	>0.05
Stimulation length (days)	10.3 ± 0.3	10.8 ± 0.1	>0.05
Number of oocytes retrieved	13.2 ± 5.9	13. 7 ± 5.5	>0.05
Endometrial thickness (HCG day) (mm)	11.0 ± 1.1	10.8 ± 2.3	>0.05
Number of fertilized oocytes	8.1 ± 4.3	8.8 ± 4.9	>0.05
Number embryos transferred	2.7 ± 0.7	2.5 ± 0.6	>0.05
Number of cleavage stage embryos	8.0 ± 3.8	8.5 ± 5.0	>0.05
CES^a ^of D3	56.4 ± 17.1	56.1 ± 16.3	>0.05
Grade I--II/all embryos transferred (%)	38/42 (90.47)	116/128(90.63)	>0.05 (90.45~99.95%)
Cancellation rates (%)	4/46 (8.69)	6/134 (4.48)	>0.05 (21.53~34.67%)
Implantation rates (%)	13/103 (12.62)	46/256 (17.97)	>0.05 (19.43%~23.77%)
Clinical pregnancy/embryo transfer (%)	11/42 (26.19)	39/128 (30.47)	>0.05 (52.33%~67.07%)
Multiple pregnancy rates (%)	2/11 (18.18%)	7/39 (17.94%)	>0.05 (95.35%~100.0%)

Values are expressed as the mean ± SEM.

ECF Group: patients with endometrial cavity fluid accumulation (equal or higher1.0 mm); Control Group: patients with bilateral tubal resection and without endometrial cavity fluid accumulation. a: CES means cumulative embryo score, defined, on the day of embryo transfer, by the multiplication of grading of fragmentation pattern of each embryo with blastomere number of the embryo to produce a single score for each embryo, and, then, by the summation of all single scores obtained from all the embryos transferred [10].

In order to investigate the impact of ECF on clinical outcome further, the level distribution of ECF in APD of the 46 patients and their cycle outcomes was analyzed and the results were shown at Table 2. Majority (97.83%) of ECF were distributed at APD 1.0 mm ~< 5.0 mm. No significant difference of ECF distribution was found at that APD interval. However, significant differences of implantation rate and clinical pregnancy rate were found between the three ECF distribution groups with their APD <3.5 mm (1.0 mm~, 2.0 mm~, and 3.0 mm~respectively) and the ECF distribution group with its APD equal or higher3.5 mm ~< 5.0 mm (P < 0.05). In the present study, the clinical pregnancy could be found in patients with their maximum ECF at APD 3.4 mm. No clinical pregnancy was found in the patients with their ECF at APD equal or higher3.5 mm.

**Table 2:** The ECF level in APD and the relevant cycle outcomes in the ECF patients

Cycle outcomes	The ECF level in APD (mm)					In total
	1.0~	2.0~	3.0~	3.5~	5.0~10.0	
Case number (%)	13/46 (28.26)	12/46 (26.09)	9/46 (19.57)	11/46 (23.91)	1/46 (2.17)	46/46 (100.0)
Cancellation rates (%)	1/13 (7.69)	1/12 (8.33)	1/9 (11.11)	1/11 (9.10)	0/1 (0.00)	4/46 (8.69)
Implantation rates (%)	6/30 (20.00)	4/24 (16.67)	3/19 (15.79)	0/28 (0.00)*	0/2 (0.00)	13/103 (12.62)
Clinical pregnancy rate/ET (%)	4/12 (33.33%)	4/11 (36.36)	3/8 (37.50%)	0/10 (0.00)*	0/1 (0.00)	11/42 (26.19)

ECF: endometrial cavity fluid; APD: the anterior--posterior diameter of endometrial cavity fluid.

*:Compared with the former 3 groups (APD 1.0 ~, 2.0 ~and 3.0 ~), X^2 ^> 3.841, P < 0.05

In order to illustrate the tendency of ECF level and the relevant cycle outcomes, all 46 cases were divided into two big subgroups by the APD of ECF as group-I: 1.0 mm < APD < 3.5 mm (n = 34) and group-II: APD equal or higher3.5 mm (n = 12). The clinical data and the cycle outcomes of the two big subgroups of ECF patients were shown as Table 3. There were no significant differences in patient characteristics between the two big subgroups. One case was cancelled her ET in the group-II because of the high risk of OHSS; while there were three cases cancelled in the group-I for the same reason. So the cancellation rates between the two subgroups were similar (8.33% versus 8.88%, X^2 ^= 0.003, P > 0.05). However, as mentioned above, the clinical pregnancy rate of the group-II was significantly lower than that of the group-I during the follow-up period (0% versus 35.48%; X^2 ^= 5.288, P < 0.05). So was the implantation rate (0% versus 17.81%; X^2 ^= 6.114, P < 0.05).

**Table 3:** Baseline and cycle outcomes in the two subgroups of ECF patients

	Group-I^a^ (n = 34)	Group-II^b^ (n = 12)	p --value (95% CI)
Age (years)	32.4 ± 0.6	32.7 ± 0.3	>0.05
Duration of infertility (years)	5.8 ± 0.4	5.6 ± 0.7	>0.05
Baseline FSH (mIU/ml)	7.4 ± 1.5	7.7 ± 1.8	>0.05
Baseline LH (mIU/ml)	5.6 ± 1.5	5.5 ± 1.7	>0.05
E_2 _level on the day of HCG (pg/ml)	2190.6 ± 1555.9	2218.5 ± 1130.8	>0.05
Stimulation length (days)	11.3 ± 0.1	10.8 ± 0.2	>0.05
Number of oocytes retrieved	13.2 ± 5.8	13.7 ± 5.3	>0.05
Endometrial thickness (HCG day) (mm)	10.0 ± 1.3	10.2 ± 1.8	>0.05
Number of fertilized oocytes	8.5 ± 3.5	8.2 ± 4.8	>0.05
Number embryos transferred	2.5 ± 0.5	2.3 ± 0.7	>0.05
No. of cleavage stage embryos	7.8 ± 3.2	8.0 ± 4.0	>0.05
CES^a ^of D3	55.8 ± 16.2	54.9 ± 16.1	>0.05
Grade I--II/all embryos transferred (%)	28/31 (90.3)	10/11 (90.9)	>0.05 (89.23~100%)
Cancellation rates (%)	3/34 (8.88%)	1/12 (8.33%)	>0.05 (90.18~100%)
Implantation rates (%)	13/73 (17.81)	0/30(0)*	<0.05 (0.18%~2.42%)
Clinical pregnancy/embryo transfer (%)	11/31 (35.48%)	0/11 (0)*	<0.05 (18.64%~52.32%)
Multiple pregnancy rates (%)	2/11 (18.18%)	0/11 (0)	>0.05 (0%~40.97%)

Values are expressed as the mean ± SEM; * X^2^>3.841, P < 0.05

a: the anterior--posterior diameter of endometrial cavity fluid<3.5 mm. b: the anterior--posterior diameter of endometrial cavity fluid equal or higher3.5 mm. c: CES means cumulative embryo score, defined, on the day of embryo transfer, by the multiplication of grading of fragmentation pattern of each embryo with blastomere number of the embryo to produce a single score for each embryo, and, then, by the summation of all single scores obtained from all the embryos transferred [10].

## Discussion

The present study showed that the presence of relatively small amount of ECF (APD < 3.5 mm) at the day of oocyte retrieval did not appear to negatively impact the clinical pregnancy rate of IVF-ET program; while the presence of excessive amount of ECF (APD equal or higher 3. 5 mm) would do. This result was similar to those described previously in some relevant studies [1,4,5]. The researchers of those studies held that ECF observed after HCG administration represents a different clinical entity and that, if the ECF was generated physiologically by the genital tract, like the ECF demonstrated in those patients without hydrosalpinx or bilateral tubal obstruction, the clinical pregnancy rate of the involved ECF patient was not worse than that of those patients without ECF and no influence of this kind of ECF on embryo implantation was found.

The time of ECF development is important in the impact of ECF on the IVF-ET outcome. Many studies demonstrated that the ECF detected during ovarian stimulation usually had a negative impact on the IVF-ET outcome [1,5,7,9]. If ECF transiently developed after receiving an HCG injection and disappeared by the day of embryo transfer, the ECF did not impact the clinical pregnancy rate [1,5]. In the present study, all ECFs were firstly observed after HCG administration and at the time of oocyte retrieval. As described in the above, no obvious impact of ECF on the IVF-ET outcome was observed, if the amount of ECF is small (APD < 3.5 mm).

The amount of ECF is also critical in the impact of ECF on the IVF-ET outcome. Among the previous relevant studies, to our knowledge, only one was conducted on the effects of ECF amount on the outcome of IVF-ET. The researchers of that study found that a large amount of ECF (>3.0 mm in the largest diameter) usually developed after receiving HCG and that the large amount of ECF was detrimental to embryo implantation [2]. In the present studies, the large amount of ECF was defined as equal or higher 3.5 mm in APD and the small amount of ECF was < 3.5 mm in APD. Both large and small amount of ECF could be found in the present study after HCG injection. The ECF less than 3.5 mm in APD usually disappeared by the time of ET; while the ECF more than 3.5 mm in APD usually persisted and enlarged until during implantation period. The presence of small amount of ECF (APD < 3.5 mm) at the day of oocyte retrieval did not appear to negatively impact the clinical pregnancy rate of IVF-ET program, when compared with that of the control patients without ECF; while the presence of excessive amount of ECF (APD equal or higher 3.5 mm) would do. To our knowledge, the present study was the first one to set up equal or higher 3.5 mm in APD of ECF as the defining value of large of fluid, such that embryo cryopreservation should be considered in patients.

Further studies are needed to be conducted to clarify the real effect of ECF on the IVF-ET outcome and the real mechanism for the development of ECF.

## Competing interests

The authors declare that they have no competing interests.

## Authors' contributions

RH carried out the design, data review and analysis of all subjects, and drafted the manuscript. HG did the same as RH, except the manuscript drafting. YL participated mainly in the data review of all subjects. XZ participated in the design and data analysis of the study, and, also, critically revised the manuscript. All authors read and approved the final manuscript.
